# Geoepidemiology, seroprevalence and factors associated with *Toxoplasma gondii* infection in domicilied cats from Paraíba (Brazil)[Fn FN1]

**DOI:** 10.1051/parasite/2024017

**Published:** 2024-05-17

**Authors:** Ana Letícia Pereira Fernandes, Mariana de Melo Alves, Jordania Oliveira Silva, Ividy Bison, Ariana de Castro Tavares Silva, Roberta Nunes Parentoni, Jose Romulo Soares dos Santos, Thais Ferreira Feitosa, Vinícius Longo Ribeiro Vilela, Arthur Willian de Lima Brasil

**Affiliations:** 1 Animal Science Program at Federal University of Paraiba – UFPB 12 Rodovia, PB-079 58397-000 Areia Paraíba Brazil; 2 Departament of Federal Institute of Paraíba – IFPB Rua Presidente Tancredo Neves, s/no, Jardim Sorrilândia 58.800-970 Sousa Paraíba Brazil; 3 Federal University of Campina Grande, UFCG Sítio Olho D’água da Bica, Zona Rural 58175-000 Cuité Paraíba Brazil; 4 Instituto de Pesquisa em Fármacos e Medicamentos of Federal University of Paraíba – UFPB, Campus I Lot, Cidade Universitária, PB 58051-900 João Pessoa Paraíba Brazil; 5 Morphology Department at Federal University of Paraiba – UFPB, Campus I Lot, Cidade Universitária 58051-900 João Pessoa Paraíba Brazil

**Keywords:** Epidemiology, Feline, IFAT, One Health

## Abstract

*Toxoplasma gondii* is a parasite responsible for toxoplasmosis, an emerging and often neglected zoonosis in South America, particularly Brazil. Felines, the only definitive hosts, excrete oocysts in their feces, potentially infecting all homeotherms. Domestic cats are primarily responsible for contaminating human environments with these oocysts. Monitoring their populations is therefore essential to ensure proper toxoplasmosis prophylaxis. The aim of this study was to estimate the prevalence of *T. gondii* and exposure factors in a population of owner cats in the city of João Pessoa, Paraíba, Brazil. A total of 267 blood samples were collected from domestic cats aged between 1 and 15 years and tested with an immunofluorescence antibody test. The seroprevalence of antibodies against *T. gondii* was only 17.22% (46/267 individuals). This result therefore suggests a low contribution of domestic cats to *T. gondii* contamination of the urban environment. The cats’ age and living environment were identified as risk factors for cat exposure to *T. gondii.*

## Abstract (fr)

*Toxoplasma gondii* est le parasite responsable de la toxoplasmose, une zoonose émergente et souvent négligée en Amérique du Sud, notamment au Brésil. Les félins, seuls hôtes définitifs, excrètent des oocystes dans leurs selles, infectant potentiellement tous les homéothermes. Les chats domestiques sont les premiers responsables de la contamination des environnements humains avec ces oocystes. La surveillance de leurs populations est donc essentielle pour garantir une prophylaxie adéquate contre la toxoplasmose. Le but de cette étude était d’estimer la prévalence de *T. gondii* et les facteurs d’exposition dans une population de chats domestiques de la ville de João Pessoa, Paraíba, Brésil. Au total, 267 échantillons de sang ont été prélevés sur des chats domestiques âgés de 1 à 15 ans et testés avec un test d’immunofluorescence des anticorps. La séroprévalence des anticorps contre *T. gondii* n’était que de 17,22 % (46/267 individus). Ce résultat suggère donc une faible contribution des chats domestiques à la contamination du milieu urbain par *T. gondii*. L’âge et le milieu de vie des chats ont été identifiés comme facteurs de risque d’exposition du chat à *T. gondii*.

## Introduction

Toxoplasmosis is a zoonosis caused by *Toxoplasma gondii*, an obligate intracellular coccidian protozoan. Felids are the only definitive hosts, since the *T. gondii* biological cycle is complete in these animals [2, 6]. Cats can excrete millions of oocysts and a single animal is capable of spreading infection to many hosts [6].

This multisystemic disease has three infective parasite forms (oocysts, tachyzoites and bradyzoites) and can be transmitted to cats mainly through ingestion of raw or undercooked meat containing cysts with bradyzoites. In humans, besides ingestion of cysts, transmission can also occur through sporulated oocysts from feces of infected cats in the environment and transplacentally (tachyzoites) [7, 23]. Risk factors associated with feline infection are sex, age, eating habits, coexistence with other species, and consumption of raw and undercooked meat and contaminated water [25].

*Toxoplasma gondii* seroprevalence in domestic cats in the world is 30–40%, and Brazil is highlighted as one of the countries with the most reports [20]. In the semi-arid region of Paraíba, a significant seroprevalence of 43.8% was observed in cats with IgG anti-*T. gondii* antibodies that presented age and hunting habits as risk factors [12]. By contrast, a seroprevalence of 26% was observed in cats from Rolim de Moura, state of Rondônia, North of Brazil, with no risk factors identified [26].

In Brazil, toxoplasmosis has a seroreactivity between 56.4% and 91.6% in women during pregnancy. Therefore, it is an important disease to One Health, especially in this group and in immunocompromized people. This disease has a great impact as it can result in miscarriage and serious neurodevelopment malformations, such as microcephaly and hydrocephalus. It is a major factor in global causes of infant morbidity and mortality [21, 28]. Furthermore, ocular toxoplasmosis is one of the relevant causes of uveitis in several countries and can explain up to 60% of cases of chorioretinitis [4].

Toxoplasmosis is an emerging, neglected zoonosis that is growing exponentially in Brazil. In urban areas, stray and domestic cats play a crucial role in transmission and maintenance of this agent as they are the only definitive hosts in direct contact with humans. Thus, health surveillance actions that assess seroprevalence and risk factors associated with feline toxoplasmosis are essential in order to obtain early diagnosis, reduce the risk of transmission, provide guidance for owners and develop prophylactic actions. For this reason, we selected a domestic cat population due to intense contact with humans and then aimed to characterize the epidemiological situation of toxoplasmosis in these cats in João Pessoa, capital of the state of Paraíba, Northeast region of Brazil.

## Material and methods

### Ethics

The present work met the standards for research involving animals in accordance with Regulation No. 38/18, which establishes restrictions on use of animals in research. The research was started only after we received a letter of approval from the Ethics Committee on the Use of Animals (CEUA) and CEP (research ethics committee) of the Federal University of Paraíba (UFPB), authorizing the research under Protocol number 3304170821. For an animal’s participation in the study, prior consent from the owner was required.

### Area, sampling and blood collection procedures

The research was carried out in João Pessoa, state of Paraíba, Brazil and was performed at clinics and owners’ houses from October 2021 to February 2022. Domestic cats of both sexes aged between 1 and 15 years were selected. Cats aged under 12 months were not included in the study due to possible interference with serological tests due to antibody levels [1].

Number of blood samples collected for the experiment was determined by simple random sampling, as follows:

$$N=\frac{Z^{2}\times p(1-p)}{d^{2}}$$

Meaning:

*N* = number of individuals sampled,

*Z* = normal distribution value for the 95% confidence level,

*p* = expected prevalence of 50%,

*d* = absolute error of 7%.

In this way, 267 feline blood samples were selected for collection. Blood collections were performed by puncturing the external jugular vein or cephalic vein using a disposable syringe and scalp with a maximum blood volume of 2 mL per cat at a clinic or the owner’s home with containment and collection according to cat friendly practices using bags, blankets and offering sachets and snacks.

The samples were stored in tubes without anticoagulant. Sera were separated from whole blood by centrifugation for 3,000 RPM at room temperature for 10 min with 80-2b Laboratory Centrifuge equipment with fixed rotor, divided into aliquots, transferred to 1.5 mL flat-bottom plastic microtubes, identified and stored at −20 °C.

### Diagnosis by indirect fluorescent antibody test

Indirect fluorescent antibody test (IFAT) was performed to diagnose toxoplasmosis. Tests were performed to detect anti-*T. gondii* (IgG) according to the technique described by [3]. For this purpose, the RH strain of *T. gondii* (tachyzoites) was fixed on glass slides. Positive and negative controls were used on each slide for monitoring. Samples that demonstrated a reaction at a 1:16 dilution were classified as positive and then diluted sequentially, in multiples of four, until maximum reactive dilution for titration with the aim of measuring the amount of antibodies in each sample [5, 11].

### Statistical analysis

In addition, a handwritten epidemiological questionnaire about the animal’s habits and health was administered orally by one interviewer to cat owners. It contained information regarding the following items and their respective categories: age (1 year/1–5 years/over 5 years), sex (female/male), street access (no/yes), castration (no/yes), type of food (pet food/homemade food/both), place where animals defecate (sandbox/yard/newspaper) and hunting habits (no/yes). These data were used to define factors associated with infection.

Analysis of factors associated with infection was divided into two moments. Firstly, a bivariate analysis was carried out where information obtained in epidemiological questionnaires (independent variables) was crossed with results of diagnostic tests (dependent variables) using chi-square or Fisher’s exact test at a confidence level of 20%. Secondly, independent variables that were significant were subjected to a multivariate analysis using Poisson regression with robust variance at a significance level of 5%. All procedures were performed in SPSS 25 for MAC.

### Georeferencing

It was decided to carry out a spatial analysis in order to verify occurrence of patterns in geographic areas by checking distribution of georeferenced points, which were obtained through addresses of owners’ homes plotted in the Google Maps app, with a level of accuracy of 20 m. After tabulating georeferenced points, a non-parametric interpolation method based on Kernel density was carried out, which made it possible to estimate distribution intensities of heat points. Kernel width (1,944 m), interpolations, spatial resolution (*X*: 79.21; *Y*: 79.21), number of rows (268) and columns (250) were automatically adjusted by ArcGis 10.4 app. Digital maps of João Pessoa are available online by the city hall and the state and federal network by IBGE.

The city’s geographic limits were plotted on a digital georeferenced map of João Pessoa, based on a map of Brazilian municipalities from 2001, obtained from the João Pessoa city hall website. Plotting and processing digital maps were carried out using ArcGIS version 9.1.

## Results and discussion

Seroprevalence of antibodies against *T. gondii* in cats was 17.22% (46/267; 95% CI = [12.7–21.8]). The titers ranged from 1:16 to 1:16,384, and the most frequent titers were 1:1,024 (9/46 animals; 19.5%) and 1:8,192 (9/46 animals; 19.5%) (Table 1).

**Table 1:** Antibody titers of anti-*Toxoplasma gondii* antibodies by IFAT of seropositive cats in the state of Paraíba, Northeast region of Brazil, in different dilutions.

Anti-*Toxoplasma gondii* antibody titers	Number of cats (%)
16	1 (2.2)
32	2 (4.4)
64	1 (2.2)
128	3 (6.5)
256	2 (4.4)
512	4 (8.8)
1.024	9 (19.5)
2.048	6 (13)
4.096	6 (13)
8.192	9 (19.5)
16.384	3 (6.5)
Total	46 (100)

The seropositivity of 17.22% demonstrates that domestic cats still have contact with *T. gondii* and produce antibodies against the protozoan. Thus, these animals play a role in toxoplasmosis epidemiology [6] since, in periods of immunosuppression, parasites that are in tissue cyst format can be reactivated and the feline can eliminate oocysts in feces [1].

Unlike other studies carried out in Northeast region of Brazil that detected prevalences of positive cats for *Toxoplasma gondii* varying between 47.7%, and 71.2%, Patos – Paraíba, and Fernando de Noronha – Pernambuco, respectively [12, 17], this research showed low seroprevalence. This possibly occurred because the studied population was made up only of domestic cats that had owners, unlike other studies where the majority of the population was stray animals. In these conditions, where owners provide care regarding nutrition, hygiene and veterinary care, there is a reduction in risk of infection [7].

Seroprevalence studies can be influenced by other factors such as the diagnostic technique used, cut-off point and where the target population lives. There are experiments that applied modified agglutination tests and indirect hemagglutination to analyze titer of anti-*T. gondii* in cats [12, 13]. In this research, ITAF was adopted as a diagnostic method since it has high sensitivity and specificity, in addition to being low cost and having specific conjugates for each species [15].

Table 2 presents the analysis of factors associated with *T. gondii* infection in cats from João Pessoa, where it was observed that the variables age (more than 60 months: RP = 5.744, CI = [1.368–24.121], *p* = 0.017) and region where they live (urban area: RP = 8.515, CI = [4.424–16.390], *p* < 0.0001) represented a significant risk.

**Table 2:** Factors associated with risk of *Toxoplasma gondii* infection in cats resulting from a univariate and multivariate analysis, from João Pessoa, state of Paraíba, Brazil, from October 2021 to February 2022.

Variables	Categories	Total	Positives (%)	*p*	Prevalence ratio	Confidence interval	*p*
Owners’ education	Elementary school	33	1 (3)	0.008*	1	–	–
	High school	87	11 (12.6)		2.915	[0.401–21.212]	0.29
	University education	147	34 (23.1)		5.465	[0.791–37.753]	0.085
Age	Up to 12 months	53	2 (3.8)	0.003*	1	–	–
	13 to 60 months	157	28 (17.8)		3.509	[0.856–14.385]	0.081
	More than 60 months	57	16 (28.1)		5.744	[1.368–24.121]	0.017**
Region where it lives	Urban area	266	45 (16.9)	0.172*	8.515	[4.424–16.390]	<0.0001**
	Urban and rural area	1	1 (100)		1	–	–
Breed	No defined breed	264	46 (17.4)	1.000	–	–	–
	With breed	3	0 (0)				
Sex	Female	163	31 (19.0)	0.332	–	–	–
	Male	104	15 (14.4)				
Street access	No	196	34 (17.3)	0.932	–	–	–
	Yes	71	12 (16.9)				
Litter box use	No	94	15 (16.0)	0.685	–	–	–
	Yes	173	31 (17.9)				
Food	Pet food	219	7 (16.9)	0.737	–	–	–
	Homemade food	2	0 (0)				
	Both	46	9 (19.6)				
Contact with other animals	No	34	8 (23.5)	0.298	–	–	–
	Yes	233	38 (16.3)				
Vaccination status	Unvaccinated	72	10 (13.9)	0.380	–	–	–
	Vaccinated	195	36(18.5)				
Contact with rodents	No	239	42 (17.6)	0.796	–	–	–
	Yes	28	4 (14.3)				
Hunting habits	No	170	28 (16.5)	0.664	–	–	–
	Yes	97	18 (18.6)				

Omnibus test: Likelihood-ratio Chi-square = 20.369; degrees of freedom = 5; *p* = 0.001. *Selected for multivariate analysis. **Factors associated with *T. gondii* infection in cats.

Animals over 60 months were 5,744 times more likely to have anti-*T. gondii*. This factor associated with risk of protozoan infection is described as classic, since older animals have a greater chance of becoming infected due to the possibility of longer exposure [24, 27].

Another factor associated with *Toxoplasma gondii* infection was that cats lived in urban areas, which were 8,515 times more likely to be positive compared to those that did not live solely in that region. Felines in urban areas possibly acquire *T. gondii* by ingesting leftover food from humans, due to low hunting availability [10, 16, 19]. Although this study targeted domestic animals, the vast majority of which tend to eat pet food, some owners reported offering both pet food and homemade food to their animals. The practice of offering raw meat without heat treatment to cats is still adopted, which may favor occurrence of infected animals, as in the environment oocysts undergo sporulation, becoming infectious and are subsequently ingested by intermediate hosts through contaminated water and food. This is the most common way for cats and humans to acquire toxoplasmosis [6].

**Figure 1:**
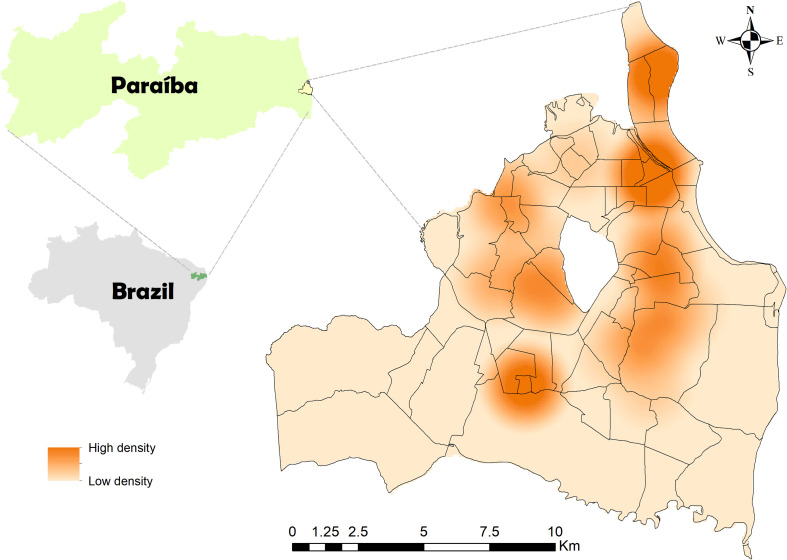
Map of the city of João Pessoa with estimated Kernel density for feline toxoplasmosis from October 2021 to February 2022.

When analyzing Figure 1 where cases of *T. gondii* are distributed on Kernel map it is observed that areas with more intense colors represent the largest clusters of seropositive animals per km^2^. Most cases were concentrated in neighborhoods in the east and south of the city, which also have the largest number of inhabitants [14]. It is important to highlight that seropositive animals will not necessarily eliminate oocysts in their feces because in any given period of time only 1% of cats are found actively excreting oocysts [9], being felines in this location unlikely to transmit toxoplasmosis to humans [8]. We believe that in João Pessoa, the environmental characteristics of high rainfall can help oocysts to survive, spread and remain accessible to potential hosts.

In all cases, handling cat feces when collecting it from a litter box should be avoided by immunocompromized people and pregnant women, given the risks and possibilities of contamination [22]. It is also necessary to pay attention to more socioeconomically vulnerable populations, which in João Pessoa are more concentrated in south and west neighborhoods [18]. In these locations, campaigns are needed to educate the population about transmission of *T. gondii*, its impacts, effects and prevention, in addition to educating about the role of domestic cats in the epidemiological chain of the disease.

## Conclusion

In conclusion, the seroprevalence of *T. gondii* infection in domestic cats in João Pessoa is considered low. However, it is important to emphasize that felines play an important role in the epidemiology of this disease. It is nonetheless suggested that cats in this research play a secondary role in transmission of toxoplasmosis. Therefore, it is crucial to reinforce information about prophylaxis and to monitor the epidemiological profile of the animal population.
